# Bioequivalence study of vonoprazan fumarate tablets in healthy Chinese subjects: a randomized, open-label, two-period crossover trial

**DOI:** 10.3389/fphar.2026.1855889

**Published:** 2026-07-10

**Authors:** Jin Yu, Sisi Lin, Yongjie Luo, Yamin Deng, Haifeng Yin, Hong Sun, Jingzhi Yu, Yiwen Zhang

**Affiliations:** 1 Center for Clinical Pharmacy, Cancer Center, Clinical Research Institute, Zhejiang Provincial People’s Hospital, Affiliated People’s Hospital, Hangzhou Medical College, Hangzhou, Zhejiang, China; 2 Center for Clinical Pharmacy, Cancer Center, I Phase Clinical Trials Ward, Zhejiang Provincial People’s Hospital, Affiliated People’s Hospital, Hangzhou Medical College, Hangzhou, Zhejiang, China; 3 Haikou Pharmaceutical Factory Co., Ltd., Haikou, Hainan, China

**Keywords:** bioequivalence, HPLC-MS/MS, pharmacokinetics, potassium-competitive acid blocker, vonoprazan fumarate

## Abstract

**Objectives:**

To evaluate the bioequivalence and safety of a test formulation (Vonoprazan Fumarate Tablets, 20 mg, T) compared with the reference product (Takecab®, 20 mg, R) in healthy Chinese subjects under both fasting and fed conditions.

**Methods:**

This was a single-center, randomized, open-label, single-dose, two-sequence, two-period, crossover study. Seventy-two healthy subjects were enrolled and assigned to either a fasting (n = 32) or a fed (n = 40) cohort. In each period, subjects received a single dose of either the test (T) or reference (R) formulation, separated by a 5-day washout period before crossing over to the alternate treatment. Plasma vonoprazan concentrations were quantified using a validated high-performance liquid chromatography-tandem mass spectrometry (HPLC-MS/MS) method. Pharmacokinetic parameters were calculated using non-compartmental analysis. Bioequivalence was assessed by determining whether the 90% confidence intervals (CIs) for the geometric mean ratios (GMRs, T/R) of C_max_, AUC_0-t_, and ${\text{AUC}}_{0‐\infty }$ fell within the predefined range of 80%–125%.

**Results:**

Seventy of the 72 enrolled subjects completed the study. Under fasting conditions, the GMRs (90% CIs) for C_max_, AUC_0-t_, and ${\text{AUC}}_{0‐\infty }$ were 95.45% (88.96%–102.41%), 98.15% (93.93%–102.56%), and 98.53% (94.41%–102.83%), respectively. Under fed conditions, the corresponding values were 105.00% (94.35%–116.85%) for C_max_, 101.96% (98.10%–105.97%) for AUC_0-t_, and 102.31% (98.58%–106.17%) for ${\text{AUC}}_{0‐\infty }$ . All 90% CIs fell within the accepted bioequivalence limits. Adverse events (AEs) were infrequent, with incidence rates of 28.1% and 25.6% in the fasting and fed groups, respectively; no serious adverse events occurred.

**Conclusion:**

The test formulation of vonoprazan fumarate is bioequivalent to the reference formulation and is well tolerated in healthy Chinese subjects under fasting and fed conditions.

**Clinical trial registration:**

https://www.chinadrugtrials.org.cn/clinicaltrials.searchlistdetail.dhtml, identifier CTR20233597.

## 1 Introduction

Gastroesophageal reflux disease (GERD) is a chronic condition characterized by the retrograde flow of gastric contents into the esophagus, causing a range of distressing symptoms and potential complications. It significantly impacts the quality of life of approximately 14% of the global population, with prevalence rates in East Asia ranging from 4.6% to 13.8% (El-Serag et al., 2014; Eusebi et al., 2018). The etiology of GERD is multifactorial, manifesting in diverse clinical presentations, of which heartburn and regurgitation are the hallmark symptoms (Fox, 2024).

Proton pump inhibitors (PPIs) have long been the gold standard for GERD pharmacotherapy. In clinical practice, however, their efficacy is often compromised by several factors, including incomplete acid suppression, suboptimal patient adherence, a delayed onset of action, and the variability associated with CYP2C19 genetic polymorphism (Kim et al., 2025). These limitations have necessitated the development of more effective acid-suppressing agents.

Vonoprazan, a novel potassium-competitive acid blocker (P-CAB), was approved in Japan in 2015 and in China in 2019 for the treatment of reflux esophagitis (RE) (Garnock-Jones, 2015). It features a distinct mechanism of action, characterized by selective accumulation within the acidic canaliculi of gastric parietal cells and the potent, reversible inhibition of the H^+^/K^+^ -ATPase pump (Wang et al., 2022). Due to its chemical stability in acidic environments and meal-independent dosing, vonoprazan offers clinical advantages such as rapid onset, sustained acid suppression, and low inter-individual variability (Xiao et al., 2020).

Pharmacokinetically, vonoprazan possesses a relatively long plasma half-life and rapidly increases intragastric pH after a single dose (Sakaguchi et al., 2024). It is primarily metabolized by CYP3A4, which potentially reduces the risk of drug-drug interactions compared to PPIs (Okubo et al., 2024). Furthermore, while it lacks direct antibacterial activity against *Helicobacter pylori* (*H. pylori*), its profound acid suppression optimizes the intragastric environment, thereby enhancing the efficacy of concomitant antibiotics for *H. pylori* eradication (Xiao et al., 2020).

As generic versions of vonoprazan enter the market, establishing their bioequivalence to the innovator product is essential to ensure therapeutic consistency. Consequently, this study evaluated and compared the pharmacokinetic profiles, relative bioavailability, and safety of a generic test formulation (T, Vonoprazan Fumarate Tablets, 20 mg) and the reference product (R, Takecab®, 20 mg) in healthy Chinese subjects under both fasting and fed conditions. Our findings aim to confirm the bioequivalence of these two formulations and provide evidence for their therapeutic interchangeability in clinical practice.

## 2 Materials and methods

### 2.1 Study design and ethics

This was a single-center, randomized, open-label, two-formulation, single-dose, two-sequence, two-period crossover study, consisting of separate fasting and fed phases (Chen et al., 2025). The protocol was approved by the Medical Ethics Committee of Zhejiang Provincial People’s Hospital (Approval No.: Zhe Ren Yi Ethics Review 2023 Drug No. 142). This study was registered at the Drug Clinical Trial Registration and Information Disclosure Platform (http://www.chinadrugtrials.org.cn) with the identifier CTR20233597. The trial was conducted in full compliance with the ethical principles of the Declaration of Helsinki, Good Clinical Practice guidelines issued by the National Medical Products Administration (NMPA), and the ICH/GCP guidelines (World Medical Association, 2024; National M edical Products Administration, 2020). All participants provided written informed consent prior to enrollment.

**FIGURE 1:**
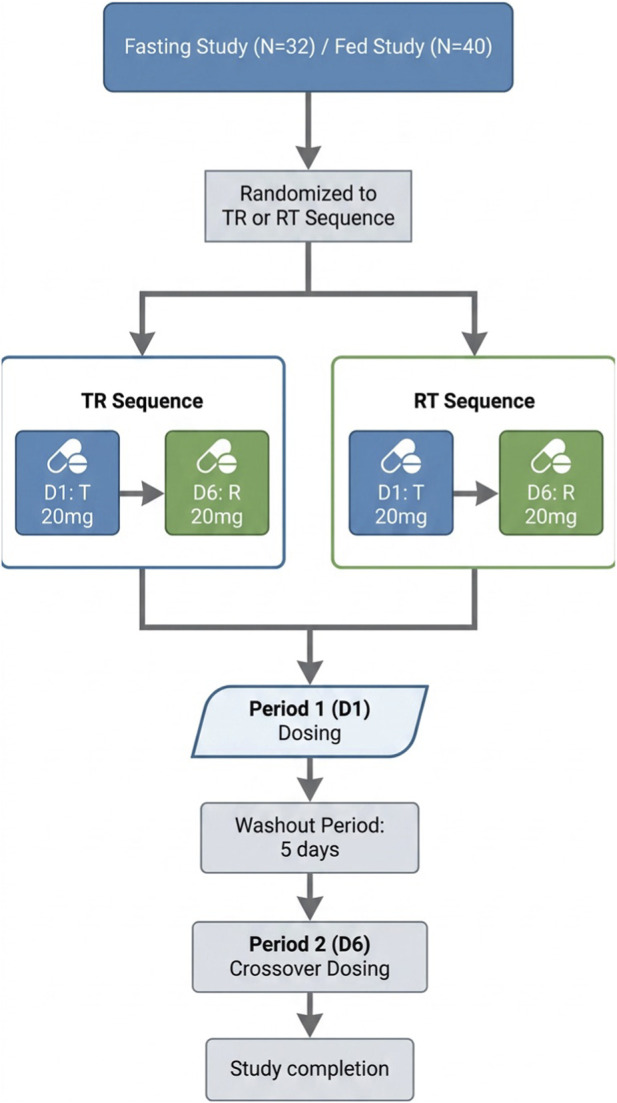
Flow chart of the bioequivalence study.

Seventy-two healthy subjects were enrolled, with 32 assigned to the fasting group and 40 to the fed group. Subjects were randomized to either the TR or RT sequence to receive a single oral dose of 20 mg of the T or R formulation under fasting or fed conditions, followed by a crossover after a 5-day washout period (Endrenyi and Tothfalusi, 2019). After successful screening, subjects were admitted to the phase I unit 2 days before dosing (Day-2), where a standardized diet was provided. On Day-1, subjects underwent an overnight fast (at least 10 h) with water permitted. In the fasting group, the study drug was administered in the fasting state on the morning of dosing. In the fed group, the drug was administered within 30 min (±0.5 min) after starting a standardized high-fat, high-calorie meal (U.S. Food and Drug Administration, 2022). A standardized lunch and dinner were provided at 4 and 10 h post-dose, respectively. The package insert for Vonoprazan Fumarate Tablets indicates a mean elimination half-life (t_1/2_) of approximately 7.0 ± 1.0 h (fasting) and 7.1 ± 0.7 h (fed) in Chinese subjects following a single oral dose (Takeda Pharmaceutical Company, 2023). Accordingly, the 5-day washout interval between treatment periods exceeds seven half-lives, which was sufficient to minimize carryover effects (Figure 1).

### 2.2 Inclusion criteria

Voluntary provision of written informed consent before any study procedures.

Healthy male and female subjects aged between 18 and 65 years (inclusive), with an appropriate gender ratio.

Body weight ≥50.0 kg for males and ≥45.0 kg for females, with a body mass index (BMI) ranging from 19.0 to 26.0 kg/m2 (inclusive).

No pregnancy plans for the subject or their partner within the next 3 months, willingness to use effective contraception throughout the study, and no plans for sperm or egg donation.

Ability to understand and comply with all study procedures and requirements.

### 2.3 Exclusion criteria

History of allergic reactions to any substance (e.g., food or drugs), or known hypersensitivity to vonoprazan or its excipients.

Presence of any clinically significant medical condition that, in the investigator’s judgment, could compromise subject safety or study evaluations, including but not limited to:

1. History of any chronic or serious disease.
2. Clinically significant abnormalities in vital signs, physical examination, 12-lead ECG, or clinical laboratory tests at screening.
3. History or surgery of any condition that may affect drug absorption, distribution, metabolism, or excretion.

History of substance abuse or alcohol dependence, or a positive alcohol breath test or urine drug screen at screening.

Specific medication and vaccination history:

1. Use of any drugs known to induce or inhibit hepatic enzymes, or specific medications such as thiazide diuretics, corticosteroids, thyroid hormones, and sympathomimetics within 28 days prior to dosing.
2. Use of any prescription drugs, over-the-counter medications, Chinese herbal medicines, or dietary supplements within 14 days prior to dosing.
3. Administration of any vaccine within 4 weeks prior to dosing.

Specific lifestyle and dietary history:

1. Regular smoking (≥5 cigarettes/day) within 3 months prior to dosing, or inability to abstain from tobacco use during the study.
2. Regular alcohol consumption (>14 units/week) within 3 months prior to dosing, or inability to abstain during the study.
3. Habitual excessive consumption of tea, coffee, or caffeinated beverages within 3 months prior to dosing, or inability to comply with restrictions.
4. Consumption of any food or beverage known to affect drug metabolism (e.g., containing caffeine, xanthine) within 48 h prior to dosing.
5. Consumption of grapefruit, orange, mango, pitaya, carambola, or their products within 14 days prior to dosing.
6. Lactose intolerance.
7. Inability to comply with the standardized diet or presence of dysphagia.

Other conditions that may interfere with the study:

1. Poor tolerance to venipuncture, or history of vasovagal syncope with needles or blood draws.
2. Participation in another clinical trial or blood loss/donation ≥400 mL within 3 months prior to dosing.
3. Pregnancy, lactation, intention of pregnancy (subject or partner) from screening until 3 months after study completion, or unwillingness to use effective, non-hormonal contraception.
4. Any other reason deemed by the investigator to make participation unsuitable.

### 2.4 Bioanalytical method

The concentration of vonoprazan in plasma was determined using high-performance liquid chromatography-tandem mass spectrometry (HPLC-MS/MS) following protein precipitation. Vonoprazan-d4 was used as the internal standard. Chromatographic separation was performed on an Agela Venusil C18 Plus column (2.1 × 50.0 mm, 5.0 μm) maintained at 35 °C. The mobile phase consisted of water containing 0.1% formic acid and 10 mM ammonium formate (mobile phase A) and acetonitrile (mobile phase B), delivered at a flow rate of 1.00 mL/min with an initial organic phase proportion of 15.0%. Detection was carried out in multiple reaction monitoring (MRM) mode with ion transitions of m/z 346.0 → 315.1 for vonoprazan and m/z 350.3 → 316.0 for vonoprazan-d4. The method demonstrated a linear range of 0.100–50.0 ng/mL, with a lower limit of quantification (LLOQ) of 0.100 ng/mL. Quality control (QC) samples were prepared at four concentration levels: 0.300 ng/mL (low QC, LQC), 2.00 ng/mL (geometric mean QC, GMQC), 15.0 ng/mL (medium QC, MQC), and 37.5 ng/mL (high QC, HQC). The method validation confirmed that accuracy, precision, selectivity, matrix effect, recovery, and stability all met the acceptance criteria outlined in the bioanalytical guidance issued by the US FDA and China NMPA (U.S. Food and Drug Administration, 2018).

### 2.5 Pharmacokinetic and bioequivalence evaluation

The pharmacokinetic parameters of vonoprazan were calculated using non-compartmental analysis (NCA). The primary endpoints included the maximum plasma concentration (C_max_), area under the plasma concentration-time curve from time zero to the last measurable concentration (AUC_0-t_), area under the plasma concentration-time curve from time zero extrapolated to infinity ( ${\text{AUC}}_{0‐\infty }$ ), time to C_max_ (T_max_), elimination rate constant (λ_z_), elimination half-life (t_1/2_), and the percentage of AUC extrapolated (AUC__%Extrap_), which was calculated as [( ${\text{AUC}}_{0‐\infty }$ - AUC_0-t_)/ ${\text{AUC}}_{0‐\infty }$ ] × 100%.

Bioequivalence was determined by comparing the geometric mean ratios (Test/Reference) and their 90% CIs for C_max_, AUC_0-t_, and ${\text{AUC}}_{0‐\infty }$ . The acceptance range for bioequivalence was set at 80%–125% (U.S. Food and Drug Administration, 2025). All pharmacokinetic calculations were performed using Phoenix WinNonlin software (version 8.3 or higher).

### 2.6 Statistical analysis and sample size

A standard bioequivalence statistical (BES) analysis was applied. The primary pharmacokinetic parameters (C_max_, AUC_0-t_, and ${\text{AUC}}_{0‐\infty }$ ) were log-transformed and evaluated using analysis of variance (ANOVA). The ANOVA model included sequence, treatment, and period as fixed effects, with subject (nested within sequence) as a random effect.

Sample size was calculated using PASS software (version 15.0) in accordance with relevant statistical guidelines for bioequivalence studies (National Medical Products Administration, 2018). The calculation employed a two one-sided tests procedure with a significance level (α) of 0.05 and a statistical power (1-β) of 0.90. This means the study had a 90% chance to correctly declare bioequivalence if the true ratio was within the 80%–125% range. The bioequivalence acceptance range was set at 80%–125%, and a 20% dropout rate was accounted for in the enrollment plan.

Specific calculations were as follows:For the fasting study, the GMR and within-subject coefficient of variation (CVw%) were assumed to be 0.96% and 21%, respectively, resulting in a minimum sample size of 25 subjects. To accommodate potential dropouts, 32 subjects were planned for enrollment. For the fed study, the corresponding assumptions were a GMR of 1.04 and a CVw% of 24%. The calculated minimum sample size was 31 subjects, with 40 subjects planned for enrollment to mitigate dropout effects. Accordingly, a total of 72 subjects were enrolled across both studies to ensure sufficient statistical power for the bioequivalence assessment.

### 2.7 Blood sampling schedule and procedures

In the fasting study, 18 blood samples were collected per period at the following times: pre-dose (within 60 min), and at 15, 30, and 45 min, and 1, 1.25, 1.5, 1.75, 2, 2.25, 2.5, 3, 4, 6, 8, 12, 24, and 48 h post-dose.

In the fed study, 19 blood samples were collected per period at: pre-dose (within 60 min), and at 15, 30, and 45 min, and 1, 1.5, 2, 2.5, 2.75, 3, 3.25, 3.5, 4, 5, 6, 8, 12, 24, and 48 h post-dose.

For each sample, 4 mL of venous blood was collected into vacuum tubes containing EDTA-K2 anticoagulant. Plasma was separated by centrifugation (4 °C, 1700 × g, 10 min) and stored at −90 °C to −60 °C pending analysis.

### 2.8 Safety evaluation

All adverse events (AEs) and serious adverse events (SAEs) occurring during the study were monitored and recorded. Concomitant medications, changes in clinical laboratory test results (including hematology, blood chemistry, and urinalysis), clinical symptoms, vital signs, 12-lead electrocardiograms (ECG), and physical examination findings were also documented. Each event or finding was assessed for severity, potential relationship to the study drug, and clinical outcome.

## 3 Results

### 3.1 Subjects

A total of 72 healthy subjects were enrolled in this study. In the fasting study, 32 subjects were enrolled from 96 screened individuals; 30 completed the trial, while two (K015 and K016, both in the R-T sequence) withdrew before the second-period dose due to non-compliance. In the fed study, 40 subjects were enrolled from 118 screened individuals; 38 completed the trial. One subject (C002, R-T sequence) was not dosed in either period due to failure to completely consume the high-fat meal within the required timeframe, and another (C006, T-R sequence) was withdrawn after receiving non-study medication for a hordeolum and did not receive the second-period dose. Baseline demographic characteristics showed no statistically significant differences between the two study groups (Table 1), confirming their comparability.

**TABLE 1:** Demographic descriptive information (Mean ± SD).

​	Fasting			Fed		
	T-R (N = 16)	R-T (N = 16)	ALL (N = 32)	T-R (N = 20)	R-T (N = 19)	ALL (N = 39)
Sex, n (%)	​	​	​	​	​	​
Male	14 (87.5%)	13 (81.3%)	27 (84.4%)	19 (95.0%)	19 (100%)	38 (97.4%)
Female	2 (12.5%)	3 (18.8%)	5 (15.6%)	1 (5.0%)	0	1 (2.6%)
Age, y	32.6 ± 9.83	27.6 ± 7.73	30.1 ± 9.05	33.1 ± 9.79	32.2 ± 7.14	32.6 ± 8.50
Height, cm	168.19 ± 8.21	166.22 ± 8.21	167.20 ± 8.14	168.93 ± 5.01	168.13 ± 5.30	168.54 ± 5.10
Weight, kg	63.29 ± 7.42	61.39 ± 6.71	62.34 ± 7.02	66.82 ± 6.23	62.96 ± 6.56	64.94 ± 6.61
BMI, kg/m^2^	22.39 ± 2.26	22.20 ± 1.37	22.29 ± 1.82	23.41 ± 1.73	22.24 ± 1.65	22.84 ± 1.77

Abbreviations: SD, standard deviation; BMI, body mass index.

### 3.2 Pharmacokinetics and bioequivalence

For the fasting study, all 32 enrolled subjects were included in the full analysis set (FAS), safety set (SS), pharmacokinetic concentration set (PKCS), pharmacokinetic parameter set (PKPS), and bioequivalence set (BES).

For the fed study, 39 subjects were included in the FAS, SS, PKCS, PKPS, and BES. This set comprised the 40 enrolled subjects, excluding subject C002 (who was not dosed in either period).

**FIGURE 2:**
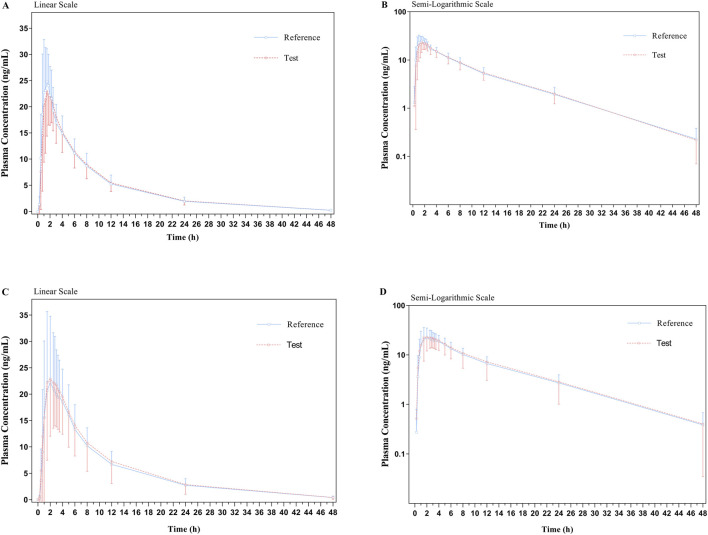
Mean ± SD plasma concentration-time curve. **(A)** Linear-scale profile following a single oral dose of vonoprazan fumarate tablets under fasting state. **(B)** Semi-logarithmic plot of the profile following a single oral dose under fasting state. **(C)** Linear-scale profile following a single oral dose under fed state. **(D)** Semi-logarithmic plot of the profile following a single oral dose under fed state.

The mean plasma concentration-time profiles of the T and R formulations were nearly superimposable under both fasting and fed conditions (Figure 2). Key pharmacokinetic parameters are summarized in Table 2.

**TABLE 2:** Pharmacokinetic parameters of vonoprazan fumarate tablets (mean ± SD (CV%)).

​	Fasting		Fed	
	T (N = 30)	R (N = 32)	T (N = 39)	R (N = 38)
T_max_^*^(h)	1.63 (0.75, 2.50)	1.50 (0.75, 2.25)	2.00 (0.75, 5.00)	2.50 (0.75, 5.00)
C_max_ (ng/mL)	27.75 ± 8.20 (29.54)	29.04 ± 8.32 (28.64)	32.52 ± 13.82 (42.48)	30.68 ± 12.10 (39.43)
AUC_0-t_ (h*ng/mL)	212.21 ± 57.69 (27.19)	211.07 ± 50.62 (23.98)	262.28 ± 112.13 (42.75)	247.75 ± 80.33 (32.42)
${\text{AUC}}_{0-\infty }$ (h*ng/mL)	216.30 ± 57.40 (26.54)	214.71 ± 50.72 (23.62)	268.83 ± 115.14 (42.83)	253.49 ± 83.08 (32.78)
λ_z_ (1/h)	0.0925 ± 0.0138 (14.90)	0.0915 ± 0.0125 (13.64)	0.0857 ± 0.0137 (16.00)	0.0851 ± 0.0132 (15.50)
t_1/2_ (h)	7.64 ± 1.03 (13.51)	7.70 ± 0.98 (12.71)	8.30 ± 1.35 (16.26)	8.34 ± 1.35 (16.14)
AUC__%Extrap_ (%)	2.17 ± 2.12 (97.70)	1.87 ± 2.19 (117.13)	2.56 ± 2.41 (94.28)	2.24 ± 1.98 (88.37)
Vd/F (L)	1092.88 ± 353.79 (32.37)	1090.93 ± 310.63 (28.47)	996.02 ± 316.43 (31.77)	1011.79 ± 237.12 (23.44)
CL/F (L/h)	100.70 ± 35.36 (35.11)	99.24 ± 28.03 (28.24)	85.56 ± 30.92 (36.13)	86.81 ± 27.04 (31.15)

T_max_^*^ was described by median (minimum, maximum).

Abbreviations: CV, coefficient of variation.

Bioequivalence analysis (Table 3) demonstrated that the 90% CIs for the geometric mean ratios (T/R) of C_max_, AUC_0-t_, and ${\text{AUC}}_{0‐\infty }$ were entirely within the predefined acceptance range of 80%–125% under both conditions.

**TABLE 3:** Bioequivalence analysis.

​	Geometric mean and ratio			90% CI (%)	CV (%)	Power (%)
	T	R	T/R (%)			
Fasting	N = 30	N = 32	​	​	​	​
C_max_ (ng/mL)	26.61	27.88	95.45	[88.96, 102.41]	16.19	99.4
AUC_0-t_ (h*ng/mL)	200.68	204.47	98.15	[93.93, 102.56]	10.03	>99.9
${\text{AUC}}_{0‐\infty }$ (h*ng/mL)	205.35	208.41	98.53	[94.41, 102.83]	9.75	>99.9
Fed	N = 39	N = 38	​	​	​	​
C_max_ (ng/mL)	30.04	28.61	105.00	[94.35, 116.85]	28.26	84.9
AUC_0-t_ (h*ng/mL)	244.34	239.65	101.96	[98.10, 105.97]	9.98	>99.9
${\text{AUC}}_{0‐\infty }$ (h*ng/mL)	250.77	245.12	102.31	[98.58, 106.17]	9.60	>99.9

Analysis of variance (Supplementary Table S1) indicated no statistically significant formulation effect for the primary parameters Ln (C_max_), Ln (AUC_0-t_), and Ln ( ${\text{AUC}}_{0‐\infty }$ ) (all P > 0.05). A statistically significant period effect was observed for some Ln (AUC) parameters (P < 0.05), which did not affect the primary conclusion of bioequivalence.

### 3.3 Safety evaluation

Safety results are summarized in Table 4. All adverse events (AEs) were mild or moderate in intensity. No serious adverse events (SAEs) or AEs leading to study discontinuation were reported. The incidence of AEs was similarly low between the test and reference formulations and remained within the known safety profile described for the reference product.

**TABLE 4:** Summary of adverse events.

​	Fasting				Fed			
	T (N = 30)		R (N = 32)		T (N = 39)		R (N = 38)	
SOC/PT	Case (%)	N	Case (%)	N	Case (%)	N	Case (%)	N
AEs	4 (13.3%)	4	5 (15.6%)	8	4 (10.3%)	12	6 (15.8%)	7
SAEs	0	0	0	0	0	0	0	0
AEs leading to exit	0	0	0	0	0	0	0	0
Various examinations	4 (13.3%)	4	2 (6.3%)	4	0	0	0	0
Gamma-glutamyl transferase increased	0	0	1 (3.1%)	1	0	0	1 (2.6%)	1
Alanine aminotransferase increased	0	0	1 (3.1%)	1	0	0	0	0
Hypertriglyceridaemia	1 (3.3%)	1	0	0	1 (2.6%)	1	1 (2.6%)	1
Hyperuricaemia	1 (3.3%)	1	0	0	1 (2.6%)	1	0	0
White blood cell count decreased	0	0	0	0	0	0	1 (2.6%)	1
Albumin urine present	0	0	1 (3.1%)	1	2 (5.1%)	2	1 (2.6%)	1
Protein urine present	0	0	1 (3.1%)	1	2 (5.1%)	2	0	0
Haematuria	1 (3.3%)	1	0	0	1 (2.6%)	1	0	0
Glycosuria	0	0	0	0	1 (2.6%)	1	0	0
Urine albumin/creatinine ratio increased	0	0	0	0	2 (5.1%)	2	0	0
Hypertension	1 (3.3%)	1	0	0	0	0	0	0
Infections and infestations	0	0	1 (3.1%)	1	0	0	0	0
Upper respiratory tract infection	0	0	1 (3.1%)	1	0	0	0	0
Asymptomatic bacteriuria	0	0	0	0	1 (2.6%)	1	1 (2.6%)	1
Hordeolum	0	0	0	0	1 (2.6%)	1	0	0
Respiratory, thoracic and mediastinal disorders	0	0	1 (3.1%)	1	0	0	0	0
Epistaxis	0	0	1 (3.1%)	1	0	0	0	0
Cardiac disorders	0	0	1 (3.1%)	1	0	0	0	0
Atrioventricular block first degree	0	0	1 (3.1%)	1	0	0	0	0
Blood and lymphatic system disorders	0	0	1 (3.1%)	1	0	0	0	0
Anaemia	0	0	1 (3.1%)	1	0	0	1 (2.6%)	1
Skin and subcutaneous tissue disorders	0	0	0	0	0	0	1 (2.6%)	1
Rash	0	0	0	0	0	0	1 (2.6%)	1

When calculating the incidence of AEs, multiple events within the same SOC, or PT, for a given subject were counted once.

Abbreviations: AE, adverse event; SAE, serious adverse event; SOC, system organ class; PT, preferred term.

## 4 Discussion

This study comprehensively evaluated the bioequivalence and safety of a test formulation of vonoprazan fumarate versus the reference product (Takecab®) in healthy Chinese subjects under fasting and fed conditions. The pivotal finding is that the two formulations are bioequivalent under both prandial states, with comparable safety profiles. These results provide a robust pharmacokinetic foundation for the clinical substitution of this generic product, which is significant for enhancing therapeutic accessibility.

The results of this study are consistent with previously reported bioequivalence studies in a Japanese population, demonstrating that the pharmacokinetic profile of vonoprazan is stable across different populations (Sugano, 2018). Recently, Japanese researchers have further elucidated the mechanistic basis underlying this observation at the metabolic level. Sakaguchi et al. found that vonoprazan is primarily metabolized by CYP3A4 into inactive metabolites, and that plasma vonoprazan concentrations are significantly correlated with CYP3A activity (Sakaguchi et al., 2024). This CYP3A4-dependent metabolic pathway—in contrast to proton pump inhibitors, which are heavily influenced by CYP2C19 genetic polymorphisms—provides a key mechanistic explanation for the predictable exposure of vonoprazan across different individuals and populations, thereby supporting the cross-ethnic robustness of the bioequivalence conclusion in the present study. The bioequivalence analysis confirmed that for all primary endpoints (C_max_, AUC_0-t_, and ${\text{AUC}}_{0‐\infty }$ ), the 90% CI fell entirely within the regulatory acceptance range of 80%–125% (U.S. Food and Drug Administration, 2025). Analysis of variance further indicated no statistically significant formulation effect (P > 0.05) for these parameters, statistically corroborating the bioequivalence conclusion (Schütz et al., 2014). A notable period effect was observed for some AUC parameters (P < 0.05), which is not uncommon in crossover designs and may stem from intra-subject variability, environmental factors, or analytical variations between periods (Chen et al., 2024). However, the key decision of bioequivalence relies on the confidence interval criterion. As the observed period effect was not formulation-specific, it does not affect the conclusion that the two formulations are bioequivalent (Lim and In, 2021; U.S. Food and Drug Administration, 2014).

The pharmacokinetic parameters observed in this study (median T_max_ 1.5–2.5 h; t_1/2_ 7.6–8.3 h) were consistent with those reported in the literature reaffirming the profile of vonoprazan as a potassium-competitive acid blocker (P-CAB) with rapid onset and a prolonged duration of acid suppression (Jenkins et al., 2015; Scarpignato and Hunt, 2024; Scarpignato et al., 2023). As anticipated, postprandial administration resulted in a delayed T_max_, a phenomenon consistent with the reported pharmacokinetics of vonoprazan and aligned with the general principle that a high-fat diet slows gastric emptying and thereby affects drug absorption (Mulford et al., 2022). A noteworthy finding was the markedly increased variability in C_max_ under fed conditions. The overall coefficient of variation increased from 29.5% in the fasted state to 42.5% in the fed state, while the within-subject coefficient of variation, which is pivotal for bioequivalence assessment, also rose from 16.2% to 28.3% (Table 3). Such food-induced increases in variability are not uncommon in bioequivalence studies and may be attributed to complex inter-individual differences in factors such as food composition, gastrointestinal motility, and bile secretion, all of which can influence drug dissolution and absorption (Karalis et al., 2012; Wang et al., 2025). Nevertheless, the crossover design and sufficient sample size employed in this study effectively controlled for this variability, ensuring the reliability of the bioequivalence evaluation (National Medical Products Administration, 2018).

All adverse events in this study were mild or moderate, primarily transient laboratory abnormalities with minimal clinical significance. The incidence of adverse events was similar between formulations, and no serious adverse events occurred. This favorable safety profile is consistent with the findings of a recent large-scale Japanese post-marketing surveillance study, which enrolled 1,174 patients receiving long-term vonoprazan maintenance therapy and reported an adverse drug reaction incidence of 2.3% with no new safety signals identified (Manabe et al., 2026). Thus, both our controlled trial and real-world evidence confirm the well-established favorable safety profile of vonoprazan.

The strengths of this study lie in its rigorous design. The randomized, open-label, two-period crossover design effectively controlled for inter-individual variability. The sample size was strictly estimated based on a power calculation, yielding high statistical power (power >99.9% for most endpoints), which ensures high reliability of the results (U.S. Food and Drug Administration, 2014). Furthermore, a sensitive and specific HPLC-MS/MS method was employed for bioanalysis, and a complete method validation was conducted in compliance with international guidelines (Fluhler et al., 2014).

This study also has limitations. First, the investigation was conducted in healthy subjects, whose gastric pH environment may differ from that of patients with GERD. However, the objective of a bioequivalence study is to compare the *in vivo* disposition of two formulations in the same system, and its conduct in a healthy population is widely accepted by regulatory authorities (U.S. Food and Drug Administration, 2014). Future studies could further evaluate clinical efficacy in patient populations. Second, this study only investigated pharmacokinetics following a single dose. Nonetheless, given vonoprazan’s relatively long half-life (approximately 7–9 h), its steady-state exposure under a once-daily dosing regimen can be reliably predicted from single-dose data based on classical pharmacokinetic principles (Laine et al., 2022; Musuamba et al., 2021). Third, the proportion of female subjects was relatively low (fasting 15.6%, fed 2.6%), which limited the ability to assess gender differences in pharmacokinetics. However, published data indicate that vonoprazan exposure has no clinically significant sex effect (Scarpignato et al., 2022).

In summary, this study provides robust evidence that the evaluated test formulation of vonoprazan fumarate is bioequivalent to the reference formulation Takecab® in healthy subjects, with a comparable safety profile. This not only fulfills the fundamental regulatory requirements for the approval of a generic drug but also offers a reliable new alternative for clinicians and patients when a faster-acting, longer-lasting acid-suppressing agent with less influence from CYP2C19 polymorphism is needed (Echizen, 2016; Abdel-Aziz et al., 2021). The availability of this generic formulation is expected to enhance clinical access to therapy and help alleviate the associated economic burden.

## 5 Conclusion

This study demonstrates that a single 20 mg oral dose of the test formulation of vonoprazan fumarate tablets is bioequivalent to the reference product (Takecab®) in healthy Chinese subjects under both fasting and fed conditions, with comparable safety and tolerability. These findings provide a scientific basis for the clinical use of the test formulation, supporting its role in improving therapeutic access and potentially alleviating the economic burden of care.

## Data Availability

The raw data supporting the conclusions of this article will be made available by the authors, without undue reservation.
