# Supplementary material for: Bioequivalence study of vonoprazan fumarate tablets in healthy Chinese subjects: a randomized, open-label, two-period crossover trial
Source: Front Pharmacol. 2026 Jul 10;17:1855889. doi: 10.3389/fphar.2026.1855889 (PMC13396015; doi:10.3389/fphar.2026.1855889)
Supplement: Supplementary file 1 [file Table1.docx]

Supplementary Material

**Supplementary table 1** multi-factor variance analysis

|  | Main Factors | ***P*** | |
| --- | --- | --- | --- |
|  |  | **Fasting** | **Fed** |
| Ln(C_max_)(ng/mL) | Sequence | 0.0603 | 0.1206 |
|  | Period | 0.7023 | 0.5762 |
|  | Preparation | 0.2700 | 0.4464 |
| Ln(AUC_0-t_)(h*ng/mL) | Sequence | 0.2927 | 0.1489 |
|  | Period | 0.0003 | <0.0001 |
|  | Preparation | 0.4754 | 0.4015 |
| Ln(AUC_0-∞_)(h*ng/mL) | Sequence | 0.2995 | 0.1724 |
|  | Period | 0.0002 | <0.0001 |
|  | Preparation | 0.5605 | 0.3059 |
